# Expression of Synj2bp in mouse liver regulates the extent of wrappER-mitochondria contact to maintain hepatic lipid homeostasis

**DOI:** 10.1186/s13062-022-00344-8

**Published:** 2022-12-01

**Authors:** Nicolò Ilacqua, Irene Anastasia, Danylo Aloshyn, Rana Ghandehari-Alavijeh, Emily Ann Peluso, Madelaine C. Brearley-Sholto, Leonardo V. Pellegrini, Andrea Raimondi, Thomas Q. de Aguiar Vallim, Luca Pellegrini

**Affiliations:** 1 Graduate Program in Neuroscience, Faculty of Medicine, Laval University, Quebec, QC Canada; 2 Mitochondria Biology Laboratory, Brain Research Center, Laval University, Quebec, QC Canada; 3 Departments of Medicine/Cardiology and Biological Chemistry, University of California, Los Angeles, CA USA; 4 Experimental Imaging Center, San Raffaele Scientific Institute, Milan, Italy; 5 Department of Molecular Biology, Medical Biochemistry and Pathology, Faculty of Medicine, Laval University, Quebec, QC Canada

**Keywords:** WrappER, Mitochondria, Inter-organelle contact, Synj2bp, Rrbp1, ApoE, MERC, MAM, Fatty acid, VLDL, Electron microscopy, NAFLD, Lipoparticles

## Abstract

**Background:**

In mouse liver hepatocytes, nearly half of the surface area of every mitochondrion is covered by wrappER, a wrapping-type of ER that is rich in fatty acids and synthesizes lipoproteins (VLDL) (Anastasia et al. in Cell Rep 34:108873, 2021; Hurtley in Science (80- ) 372:142–143, 2021; Ilacqua et al. in J Cell Sci 135:1–11, 2021). A disruption of the ultrastructure of the wrappER-mitochondria contact results in altered fatty acid flux, leading to hepatic dyslipidemia (Anastasia et al. 2021). The molecular mechanism that regulates the extent of wrappER-mitochondria contacts is unknown.

**Methods:**

We evaluated the expression level of the mitochondrial protein Synj2bp in the liver of normal and obese (*ob/ob*) mice. In addition, we silenced its expression in the liver using an AAV8 vector. We coupled quantitative EM morphometric analysis to proteomics and lipid analyses on these livers.

**Results:**

The expression level of Synj2bp in the liver positively correlates with the extent of wrappER-mitochondria contacts. A 50% reduction in wrappER-mitochondria contacts causes hepatic dyslipidemia, characterized by a gross accumulation of lipid droplets in the liver, an increased hepatic secretion of VLDL and triglycerides, a curtailed ApoE expression, and an increased capacity of mitochondrial fatty acid respiration.

**Conclusion:**

Synj2bp regulates the extent of wrappER-mitochondria contacts in the liver, thus contributing to the control of hepatic lipid flux.

**Supplementary Information:**

The online version contains supplementary material available at 10.1186/s13062-022-00344-8.

## Background

The western diet is known to shift immune cell balance, promote cancer progression, and be at the roots of multiple problems in human health during aging, from metabolic syndrome and systemic inflammation to neuroinflammation and neurodegeneration [4–7]. The western diet is rich in triglycerides which, once ingested, are processed by gastrointestinal lipases to be assimilated as fatty acids (FA). Thus, a triglyceride-rich diet, causes a large flux of dietary FA in the cell types responsible for their assimilation, namely the enterocyte of the small intestine and the hepatocyte of the liver. Given the hydrophobic nature of FA, enterocytes and hepatocytes must, therefore, efficiently compartmentalize and traffic large quantities of dietary FA through specialized cellular structures whose characterization, however, remains largely uncharacterized.

The intracellular amount of FA is controlled through specific intracellular and systemic FA-elimination programs; together, these mechanisms prevent FA accumulation and the associated cytotoxic effects, which include activation of ER stress responses [8]. In hepatocytes, a major intracellular FA-elimination program consists in increasing FA respiration in mitochondria and peroxisomes. A second major FA-elimination program is systemic and consists in secreting the FA bound to lipocalins and albumin into the bloodstream [1, 9]. A third one is a hybrid intracellular-systemic program. It consists in condensing FA to glycerol to synthesize triglycerides which, in turn, can be temporarily accumulated intracellularly as lipid droplets (LD) or can be secreted into the bloodstream as very low-density lipoprotein (VLDL) [10, 11]. The structural basis of the circuit that distributes dietary FA to the organelles that respire, store, and secrete them remains poorly understood.

**Fig. 1:**
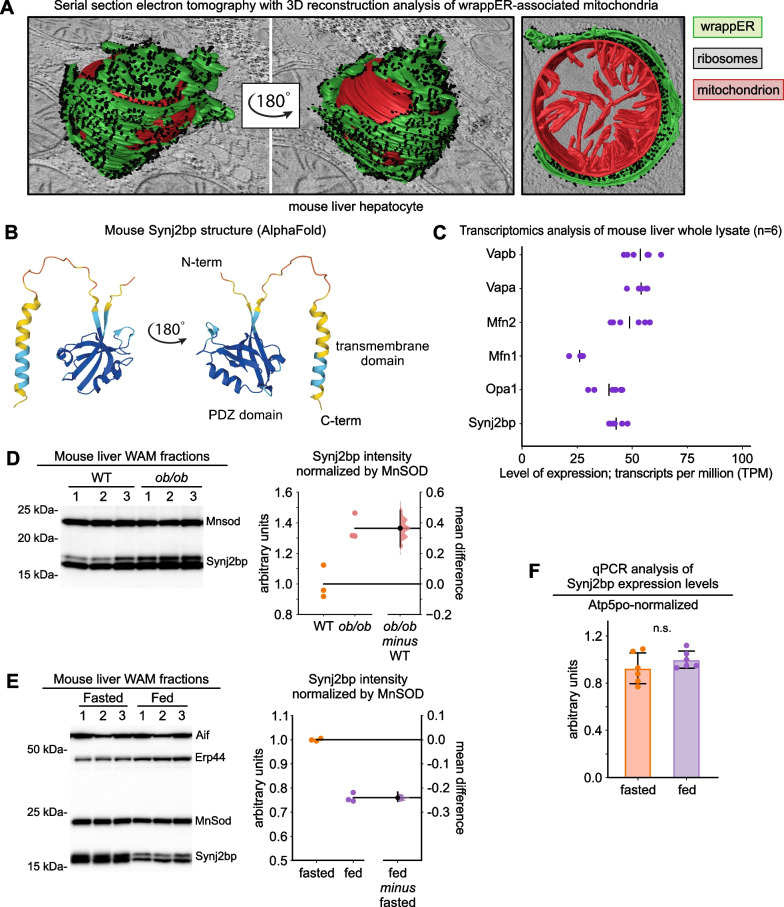
Synj2bp expression level in the liver is dynamic and shifts in response to changes in nutritional status and obesity. **A** Representative image of a wrappER-associated mitochondria (WAM) found in mouse liver hepatocytes. These images were obtained from serial section electron tomography and 3D reconstruction analysis [1]. **B** AlphaFold-predicted 3D structure of mouse Synj2bp. **C** Transcriptomic analysis of mouse liver showing the expression level of genes whose products participate in intermembrane tethering (data collected from [1]). **D**, **E** Anti-Synj2bp immunoblot and densitometry analyses (data analyzed by estimation statistics [24, 25]). **F** Synj2bp expression level in the liver of fasted (14 h) and fed (3 h postprandial) mice measured by quantitative real-time PCR analysis (*p* value calculated with Mann–Whitney test)

We recently discovered a cellular compartment that functionally and structurally integrates all the systemic and intracellular FA-elimination pathways of the liver cell. We named this compartment wrappER because it is a type of ER that, in mouse liver hepatocytes, extensively wraps around nearly every FA-respiring organelles of the cell, the mitochondrion (Fig. 1A) and the peroxisome [1–3]. Compared to other types of ER, the wrappER contains more FA and more FA-binding proteins of the lipocalin family, the MUPs, which the liver secretes into the bloodstream to regulate lipid homeostasis; furthermore, the wrappER synthesizes a substantial portion of the VLDL produced by the liver [1]. Therefore, the wrappER and its contacts with mitochondria and peroxisomes possess all the structural and functional characteristics that are expected of a regulator of the amount of dietary FA present in the liver cell. The molecular mechanisms regulating the biogenesis and dynamics of wrappER contacts with mitochondria and peroxisomes have started to emerge [1, 3, 12, 13], but their contribution to regulating intracellular FA level and flux remains largely unclear.

Our studies showed that this inter-organelle association, which can include also the peroxisome [3], is dynamic, in that it responds to changes in hepatic metabolism [1]. Indeed, the extent (i.e., the percentage) of the mitochondrial perimeter covered by the wrappER decreases, on average, from 57 to 48% within 3 h from refeeding [1]. As this happens, however, the distance of the wrappER-mitochondria contact remains unchanged at about 54 nm [1]. Neither does this value change in livers subjected to acute genetic ablation of *Mttp*, which causes acute dyslipidemia due to the abrogation of VLDL synthesis [1, 14]. Therefore, the mechanisms regulating the extent of wrappER-mitochondria contacts appear to be differentially regulated from those that control the distance of these contacts, which is regulated by Rrbp1, an ER membrane protein that AlphaFold predicts to form an extremely elongated, rigid rod-like structure that spans a distance of approximately 45 nm [15] (Additional file 1: Fig. S1A). Hepatic fractions enriched in wrappER-associated mitochondria (WAM) contain a large amount of Rrbp1 transcript and protein, which localizes at the interface of the contact between wrappER and mitochondria [1]. We found that knocking down 75% of hepatic Rrbp1 expression disrupts the uniform juxtaposition of the wrappER with the mitochondrial surface, causing the formation of wrappER "bumps" that resemble the folds of a poorly laid carpet on the ground [1]. Together with the rod-like structure and size of Rrbp1, these results converge on assigning this protein a role in regulating the distance of wrappER-mitochondria contact. Importantly, Rrbp1 silencing, while altering the distance of the wrappER-mitochondria contact, does not cause a decrease in the percentage of mitochondrial surface area covered by the wrappER, that is, does not decrease the extent of the wrappER-mitochondria contact. Thus, the nature of the regulator(s) of the extent of the wrappER-mitochondria contact remains to be determined.

Recent Bio-ID studies showed that Rrbp1 interacts with Synj2bp/OMP25 [16, 17], a mitochondrial membrane protein localized on the outer membrane of the organelle [18, 19] (Fig. 1B). Overexpression of Synj2bp in COS 7 cells increases ﻿the median percentage of mitochondrial surface in contact with ER from 6 to 25% per mitochondrion [16]. Conversely, Synj2bp silencing reduced ER-mitochondria contacts in iPS cells [13], although its knockout in HEK 293 T and HeLa cells had no apparent effects on this inter-organelle contact [16]. The role of Synj2bp in cell metabolism and animal physiology remains obscure. In this study, we found that Synj2bp regulates the extent of the wrappER-mitochondria contacts in the liver and, through this function, hepatic FA flux.

## Results

### Synj2bp protein level correlates with the extent of wrappER-mitochondria contacts in mouse liver

To investigate the role of *Synj2bp* as a regulator of wrappER-mitochondria contacts in the liver (Fig. 1A, B), we started by confirming its expression in our mouse liver transcriptomics and proteomics databases [1]. Here, we also compared the expression level of the *Synj2bp* mRNA with that of genes that regulate the biogenesis of other inter-organelle contacts in the liver. This analysis showed that the number of *Synj2bp* TPM (transcripts per million) was similar to that of *Mfn1/2* and *VapA/B* (Fig. 1C), which regulate ER-mitochondria and ER-peroxisome association in the liver, respectively [12, 20–23]. We then investigated whether the level of expression of Synj2bp in the liver correlated with the extent of wrappER-mitochondria contacts in this tissue. We started by comparing by quantitative immunoblotting the expression level of Synj2bp in the livers of control and *ob/ob* mice and analyzed the resulting data by estimation statistics [24, 25]. We showed that Synj2bp expression was significantly higher in obese animals (Fig. 1D), where wrappER-mitochondria contacts in the liver are known to be chronically enriched [26]. Further, we compared the level of expression of Synj2bp in the liver of fasting (14 h) and fed mice (3 h postprandial). These conditions were chosen because, in the fasting state, livers contain about 20% more wrappER-mitochondria contacts than in the fed state [1]. Here we found that fasting livers also contain considerably more Synj2bp (Fig. 1E), therefore establishing a direct correlation between the level of expression of this protein and the extent of wrappER-mitochondria contacts. This finding prompted us to investigate whether physiological changes in the extent of wrappER-mitochondria contacts were driven by changes in Synj2bp expression at the transcript or at the protein level. To this end, we analyzed *Synj2bp* expression by RT-qPCR using total mRNA samples acquired from livers of fasting and fed mice (n = 6 per group). We found that whereas Synj2bp protein expression decreases twofold within 3 h from feeding (Fig. 1E), the level of expression of its mRNA remains unchanged (Fig. 1F). This finding indicates that the elimination of wrappER-mitochondria contacts resulting from the fasting-feeding transition [1] occurs in parallel with the elimination of Synj2bp protein from mitochondria. We conclude that the level of hepatic expression of the Synj2bp protein positively correlates with the extent of wrappER-mitochondria contacts in the liver and that the dynamics of this inter-organelle association might impinge on the mechanism regulating Synj2bp degradation.

### Synj2bp silencing reduces the extent, but not the distance, of the wrappER-mitochondria contact

**Fig. 2:**
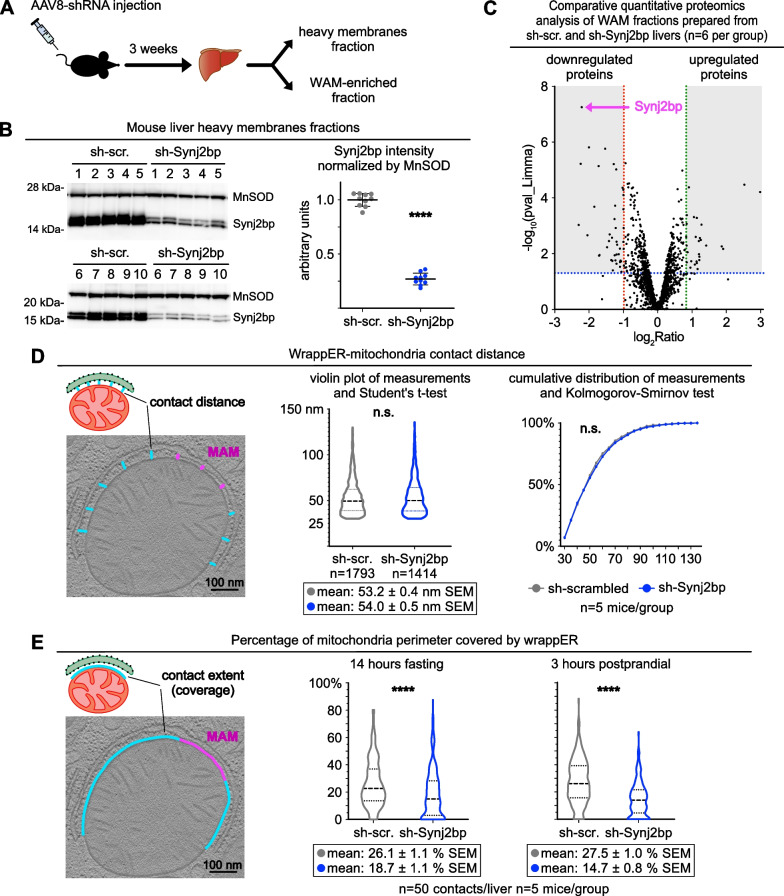
Synj2bp silencing reduces the extent, but not the distance, of the wrappER-mitochondria contact. **A** Schematic representation of the protocol used in this study. **B** Immunoblot and densitometry analysis showing Synj2bp silencing in the livers of mice injected with AAV8-shSynj2bp. **C** Comparative quantitative proteomic analysis. The volcano plot shows the proteins that are down-and up-regulated in WAM-enriched fractions isolated from Synj2bp-silenced livers compared to control AAV8-shScrambled injected mice. **D**, **E** Electron microscopy morphometric analyses of the wrappER-mitochondria contact distance (**D**) and the percentage of mitochondria perimeter covered by the wrappER (**E**) in control and Synj2bp-silenced mouse livers. Note that MAMs (magenta) were excluded from these analyses. The Student’s *t* test was used to calculate *p* values. Data were obtained from mouse livers at 3 h postprandial unless otherwise indicated. WAM: wrappER-associated mitochondria

To explore the role of Synj2bp as a regulator of the extent of the wrappER-mitochondria contact in the liver, we started by knocking down Synj2bp expression in this tissue (Fig. 2A). Quantitative immunoblot and proteomics analysis showed that AAV8-sh-Synj2bp reduces Synj2bp protein expression by 75% within three weeks of virus transduction (Fig. 2B, C and Additional file 2: Table S1). We then performed a quantitative EM analysis on the livers, focusing on wrappER-associated mitochondria (WAM), as we did in our previous studies [1, 3]. First, we investigated the effect of Synj2bp knockdown on the mitochondrial population and detected a 17% reduction in the number but a 22% increase in the average size (perimeter) of the mitochondria (Additional file 1: Fig. S1B, C). Thus, the mitochondrial mass remains unchanged, which is also confirmed by comparing the expression level of MnSOD in control and Synj2bp-silenced livers. (Fig. 2B). Second, we studied the effect of Synj2bp knockdown on wrappER-mitochondria contact proximity. To this end, we carefully measured the distance separating the two organelles in high-magnification, high-resolution EM images of approximately 1400 WAM per condition, which were taken from 5 control and 5 Synj2bp-silenced livers (Fig. 2D, left panel, blue lines). This analysis showed that Synj2bp knockdown does not change the average distance of the wrappER-mitochondria contact which, as previously reported [1], remains at about 54 nm (Fig. 2D). Finally, in the same set of EM images, we assessed the extent of the wrappER-mitochondria contact by measuring the percentage of mitochondrial perimeter covered by wrappER in 250 randomly selected WAM (Fig. 2E, left panel, blue line). Note that the MAM is excluded from these analyses (Fig. 2D, E left panel, magenta lines) because the MAM is a functionally and structurally distinct subdomain of the wrappER-mitochondria contact with unique functional properties and proteome [1, 27, 28]. This quantitative EM morphometric analysis showed that Synj2bp knockdown caused about 53% loss of wrappER-mitochondria contacts (Fig. 2E). These data thus validate the direct correlation observed between the level of hepatic expression of Synj2bp and the extent of the wrappER-mitochondria contacts present in mouse liver (Fig. 1D–E) [1, 26]. Overall, therefore, these analyses provide in vivo evidence that Synj2bp is a *bona fide* regulator of wrappER-mitochondria contacts [13, 16].

### Altered triglycerides accumulation and secretion in Synj2bp-silenced livers

A large body of previous work suggests that the contact between rough-ER and mitochondria directly contributes to the regulation of the fatty acid flux in the liver and other tissues [1, 26, 29–31]. Therefore, to validate the role of *Synj2bp* as a regulator of this inter-organelle association, we investigated whether and how its silencing altered hepatic lipid homeostasis.

**Fig. 3:**
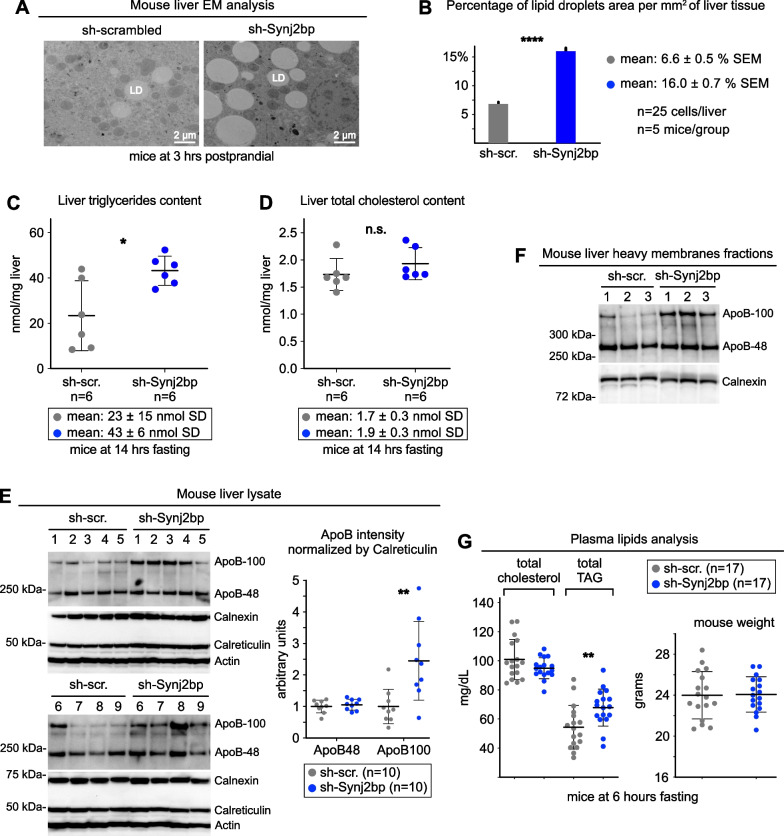
Synj2bp silencing in mouse liver increases hepatic and plasma triglycerides content. **A**, **B** Quantitative EM morphometric analyses of lipid droplets (LD) in control and Synj2bp-silenced livers (*p* values calculated with Student’s *t* test). **C**, **D** Lipid content analysis in control and Synj2bp-silenced livers. **E**, **F** Immunoblot and densitometry analysis showing changes in ApoB expression in Synj2bp-silenced livers. **G** Changes in lipid content in the plasma of mice injected with control (AAV8-shScrambled) and Synj2bp-silencing vectors (AAV8-shSynj2bp). The right panel shows the weight of the mice that were used for this analysis. Mann–Whitney test was used to calculate *p* values unless otherwise indicated

We started by measuring the level of hepatic intracellular accumulation of neutral lipids. First, we measured by quantitative EM morphometric analysis the amount of LD, whose area doubled in Synj2bp-silenced livers (Fig. 3A, B). Second, we quantified liver triglycerides, which also doubled upon Synj2bp knockdown (Fig. 3C). In contrast, as expected, total cholesterol content remained unchanged (Fig. 3D) because the amount of this lipid in the liver mostly depends on its quantity in the diet of the animal [32].

Next, we compared neutral lipids hepatic secretion in control and Synj2bp-silenced livers. Immunoblot densitometry analysis showed that Synj2bp knockdown caused a twofold increase in the expression of ApoB-100 (Fig. 3E, F), a VLDL marker and a proxy of the amount of neutral lipids that the liver can secrete into the bloodstream [33, 34]. Then, we compared the amount of circulating neutral lipids in the serum of control mice versus that in mice with Synj2bp knockdown in the liver. Consistent with the previous findings, we found that the content of triglycerides, but not cholesterol, increased by about 25% in the plasma of mice with Synj2bp-silenced livers (Fig. 3G).

**Fig. 4:**
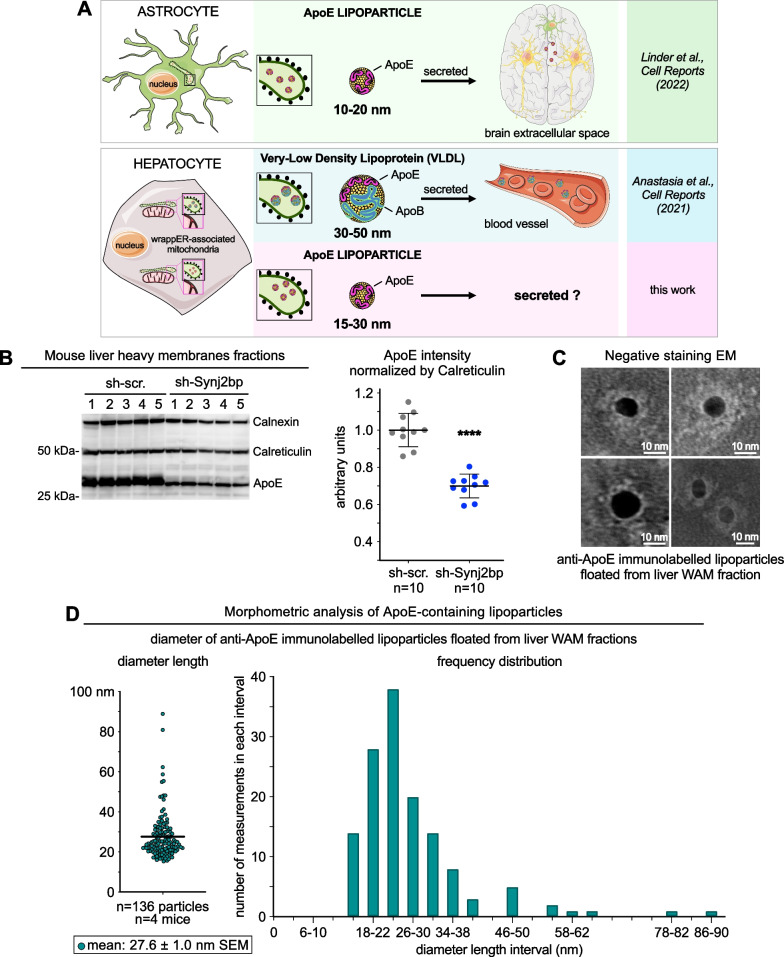
The wrappER contains small ApoE-containing lipoparticles. **A** Schematic diagram illustrating the types of ApoE-containing lipoparticles produced in the ER lumen of astrocytes and hepatocytes. **B** Immunoblot and densitometry analysis showing changes in ApoE expression in Synj2bp-silenced livers. Mann–Whitney test was used to calculate *p* value. **C** Negative staining EM coupled to immunogold labeling of anti-ApoE lipoparticles floated from mouse liver WAM-enriched fractions. **D** Size of ApoE-containing lipoparticles isolated from WAM-enriched fractions. The value of this parameter is expressed in nm and refers to the diameter of the lipoparticle. The right graph shows the frequency distribution of lipoparticle sizes. Gold particles diameter = 10 nm

We also sought evidence of perturbed FA flux by analyzing the expression level of ApoE, a key component of VLDL and, in astrocytes, a protein that assembles small lipoparticles (10–20 nm in diameter) that do not contain ApoB but are secreted extracellularly and respond to cellular stress [35] (Fig. 4A upper panel). Here, quantitative anti-ApoE immunoblot analysis showed that Synj2bp knockdown reduced ApoE expression by approximately 50% (Fig. 4B, Additional file 1: Fig. S3C), a finding that is consistent with studies showing that pathophysiological conditions change the rate of ApoE lipoparticles secretion by the brain [35]. This result prompted us to investigate whether the wrappER lumen, in addition to VLDL [1], contains also small ApoE-containing lipoparticles. To this end, we gently broke WAM fraction membranes by freezing and thawing, and floated lipoprotein particles using very high *g* forces. Then, we analyzed the isolated particles by negative staining coupled with anti-ApoE immunogold EM analysis [1, 36]. This experiment identified particles decorated with anti-ApoE that, for the vast majority, are between 15 and 30 nm in diameter (Fig. 4C, D); note that we have never observed anti-ApoB immunogold staining of lipoparticles smaller than 25 nm (Additional file 1: Fig. S2A, B), indicating that these are the hepatic counterpart of small ApoE-containing lipoparticles synthesized in the brain [35]. Whether these new types of liver lipoparticles are also secreted remains to be determined (Fig. 4A).

Finally, we observed a reduced systemic elimination capacity of FAs. Specifically, quantitative proteomics analysis of WAM fractions isolated from Synj2bp-silenced livers revealed decreased expression of Mups (Additional file 1: Fig. S3C), indicating a reduced ability to secrete FAs into the bloodstream.

Altogether, these results indicate that Synj2bp-dependent loss of wrappER-mitochondria contacts in the liver is accompanied by intracellular and systemic alterations of the most distinctive processes involved in lipid flux in various tissues and that, in the liver, are typically associated with NAFLD pathogenesis [37]. This Synj2bp phenotype agrees with that observed in Rrbp1-silenced livers, where the proximity but not the extent of the wrappER-mitochondria contact is substantially altered [1]. Collectively, these results provide further in vivo evidence in support of the notion that the contact between rough-ER and mitochondria is a key structural feature of cells dedicated to the regulation of the intracellular FA content and flux.

### Synj2bp knockdown in the mouse liver increases wrappER-mitochondria adhesion sites

**Fig. 5:**
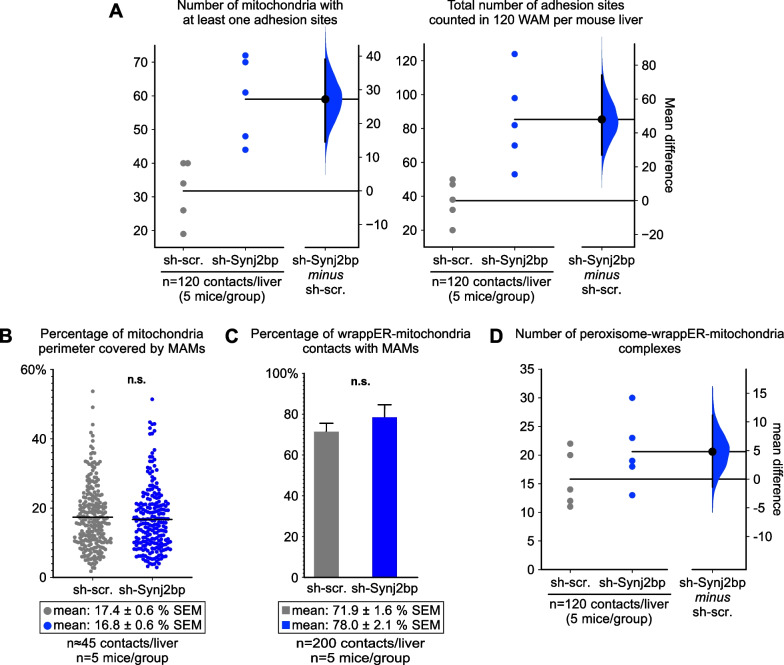
Synj2bp silencing in the mouse liver increases wrappER-mitochondria adhesion sites without affecting MAM content. **A** Quantitative EM morphometric analysis of wrappER-mitochondria adhesion sites. Here we counted the adhesion sites between the wrappER and the mitochondrial outer membrane in high-magnification EM images of wrappER-associated mitochondria (WAM) and processed the data by estimation statistics analysis [24, 25]. The graph shows that Synj2bp silencing increases the number of mitochondria with at least one adhesion site (left) and the number of adhesion sites counted in 120 WAM per liver (right; total n = 600 WAM; n = 5 mouse per condition). **B**, **C** Quantitative EM morphometric analyses of the MAMs present in wrappER-associated mitochondria. In **B**, each dot represents the length of a MAM expressed as a percentage of the perimeter length of the mitochondrion to which it belongs (measured in nm). This study shows that Synj2bp silencing changes neither the size of each MAM measured (**B**) nor the number of MAMs present in all WAMs analyzed (**C**). The Student’s *t* test was used to calculate *p* values. **D** Quantitative EM morphometric analysis of peroxisome-wrappER-mitochondria (PEWM) complexes in control and Synj2bp-silenced livers. Here we counted the PEWM complexes in high-quality EM liver images and processed the data by estimation statistics analysis. The graph shows that the number of PEWM complexes does not significantly change. Data were obtained using mouse livers at 3 h postprandial

We showed that the wrappER-mitochondria contact encompasses two structurally distinct subdomains, the MAM [38, 39] and the adhesion site [1]. The function of the adhesion sites remains to be determined, but it is likely linked to lipid flux regulation. Indeed, in *Mttp*^*−/−*^ livers [1], where VLDL synthesis is blocked [14, 40], the size and number of adhesion sites doubled despite the fact that the amount of the mitochondrial surface area covered by wrappER did not change [1]. The accumulation of adhesion sites thus seems to be triggered by the shift in fatty acid and cholesterol flux caused by the loss of VLDL synthesis. This connection suggests that the formation of the adhesion sites occurs through a process independent of that which regulates the amount of mitochondrial surface area covered by the wrappER, and which we have shown here to be under the control of Synj2bp. Consequently, because Synj2bp knockdown impairs hepatic lipid flux, it should be expected that the number of adhesion sites would increase despite the halving of the extent of contact between wrappER and mitochondrion. To test this prediction, we measured the frequency with which adhesion sites were present within the wrappER-mitochondria contact. As expected, we found that the number of adhesion sites doubled in Synj2bp-silenced livers compared with control livers (Fig. 5A). In contrast, MAM quantity and distance did not change (Fig. 5B, C and Additional file 1: Fig. S3A, B). The lack of change in MAM is also expected because the MAM, which regulate phospholipids and calcium exchanges between the ER and mitochondria [38, 39, 41], did not change in *Mttp*^*−/−*^ livers either [1].

Importantly, Synj2bp knockdown also tends to increase the number of peroxisome-wrappER-mitochondria (PEWM) complexes (Fig. 5D). These are three-organelle associations that the wrappER organizes by bringing a peroxisome and a mitochondrion into close proximity [3]. The function of the PEWM complex is not known, but it also appears to be associated with maintaining hepatic lipid homeostasis. Indeed, fasted mouse livers, which are subjected to large FA flux, contain twice as many PEWM complexes as fed livers [3]. Thus, the trend for an increase in the number of PEWM complexes observed in Synj2bp-silenced livers (Fig. 5D) is in line with previous observations [3]. Altogether, these changes in adhesion sites and PEWN complexes observed in Synj2bp-silenced livers, along with the results on wrappER-mitochondria contacts described above, support the major role of the contacts between rough-ER and FA-respiring organelles in the regulation of intracellular FA content and flux.

### Upregulated mitochondria fatty acid respiration in Synj2bp-silenced livers

**Fig. 6:**
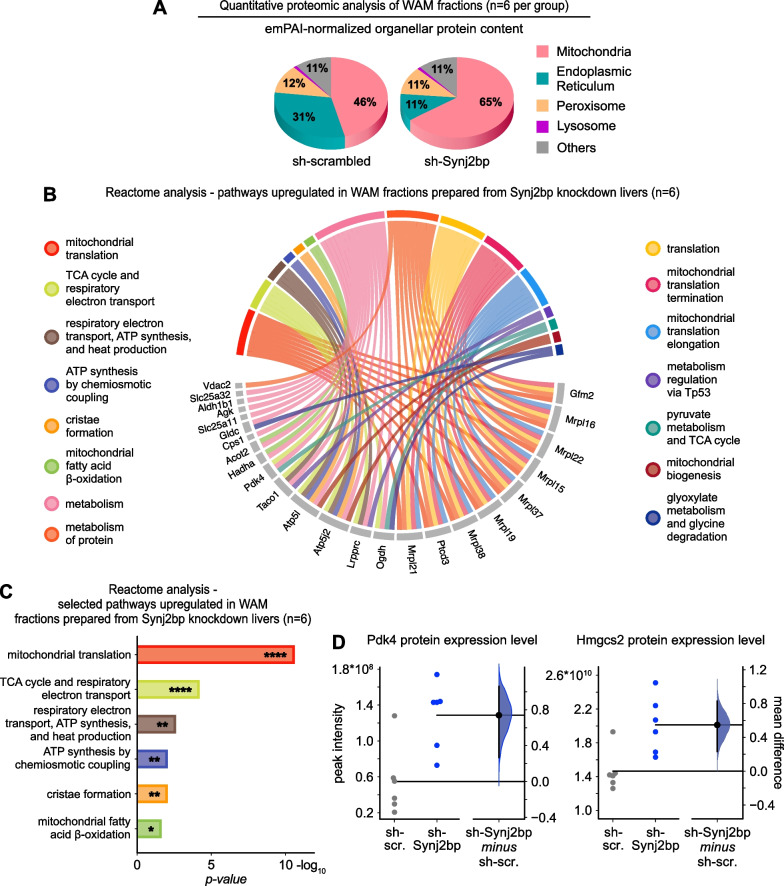
Synj2bp silencing in the mouse liver upregulates mitochondria fatty acid respiration capacity. **A**–**D** Comparative quantitative proteomic analysis of WAM-enriched fractions prepared from livers of mice injected with control AAV8-shScrambled and AAV8-shSynj2bp (livers collected at 3 h postprandial). **A** The graph shows that silencing Synj2bp, which reduces the amount of wrapper associated with mitochondria (Fig. 2E), reduces the percentage of ER proteins but proportionally increases only the amount of mitochondrial proteins, not that of other subcellular compartments. **B**, **C** Reactome biological process analysis of the pathways up-regulated in Synj2bp-silenced mouse livers. **D** Estimation statistics analysis [24, 25] of the WAM proteomics data related to the indicated proteins. Pdk4 and Hmgcs2 up-regulation in Synj2bp-silenced livers indicates activation of mitochondrial FA respiration

To gain insights into the effects of Synj2bp knockdown in hepatic metabolism, we performed quantitative proteomics analysis of the liver fractions enriched in wrappER-associated mitochondria (Fig. 2C and Additional file 2: Table S1) [42]. Compared to control fractions, WAM isolated from Synj2bp-silenced livers contained nearly half the amount of ER proteins (Fig. 6A). This data, therefore, provides biochemical evidence that abrogation of Synj2bp expression halves the amount of wrappER that is in contact with the mitochondrial population (Fig. 2E). Interrogation of the Reactome database with the set of proteins upregulated in WAM fractions isolated from Synj2bp-silenced livers showed higher translation of mitochondrial DNA-encoded proteins, which participate in the respiration of pyruvate and FA (Fig. 6B, C, Additional file 1: Fig. S3C–F). Furthermore, we observed a twofold increase in the level of expression of two mitochondrial markers of fatty acid respiration, namely Pdk4, which inhibits pyruvate respiration (Fig. 6D, left panel)  [43], and Hmgcs2, which is involved in the fate-committing step of hepatic ketogenesis (Fig. 6D, right panel) [45]. Altogether, these findings provide biochemical evidence that Synj2bp-induced loss of wrappER-mitochondria contacts upregulates the FA respiration capacity of the mitochondria. This observation parallels the trends observed in mouse of *Mttp*^*−/−*^ livers [3]. Therefore, taken altogether, these findings indicate that both the proximity [1] and extent of wrappER-mitochondria contacts (this work) are necessary for the proper regulation of FA flux within the hepatocyte.

## Discussion

In this work, we provide the first analysis of Synj2bp gene expression in vivo. Specifically, we compared the expression of the Synj2bp mRNA and protein in mouse livers collected from animals fasted for 14 h and those fed ad libitum for 3 h after fasting. Under these two conditions, the expression level of the Synj2bp transcript does not change; however, the amount of the Synj2bp protein halves within 3 h of feeding (Fig. 1E, F), indicating that posttranslational programs, but not transcriptional ones, regulate Synj2bp-related function(s) under steady-state conditions. However, this might not be the case in pathophysiological conditions because the amount of Synj2bp mRNA is fourfold higher in the post-mortem spinal cord tissue of patients with motor neuron disease than in healthy individuals [13]. At the protein level, changes in Synj2bp expression also appear to correlate with pathophysiological conditions. This possibility emerges from immunoblot analysis data showing a 40% increase in Synj2bp in obese livers (Fig. 1D) and a twofold increase in spinal cord tissue from patients with motor neuron disease [13].

Future explorations of the role of Synj2bp in human diseases will need to keep two key points into consideration. The first is to analyze the transcript and protein expression levels of Synj2bp simultaneously. The second point is to monitor the stoichiometric ratio of the expression levels of the two forms of the Synj2p protein. Immunoblot analysis of murine and human samples shows that the Synj2bp protein is expressed in two forms of slightly different molecular weights, 16 and 17 kDa, respectively (Fig. 1D, E). Typically, the 16-kDa form is most abundant, being expressed about twofold higher than the 17-kDa form (Fig. 1D, E). The two forms result from post-translational modifications because the expression of a Synj2bp cDNA generates both [13, 16]. In this study, we sought to understand whether these two bands are the product of possible phosphorylation by means of the lambda-protein phosphatase enzymatic assay. This analysis showed no evidence of Synj2bp phosphorylation (Additional file 1: Fig. S4C). The identity of these two forms of Synj2bp, therefore, remains to be discovered, but the rising importance of this protein as a regulator of mitochondrial RNA-binding activities [44, 46] and of mitochondria-ER contacts biogenesis [13, 16] (Fig. 2E), call for their prompt structural and functional characterization.

All major studies on Synj2bp agree that it is an integral membrane protein inserted into the outer membrane of the mitochondrion [18, 19, 46]. Human and mouse Synj2bp are 145-long amino acid proteins containing a predicted transmembrane helix at the C-terminus 120–139 [47]. In Synj2bp orthologs from birds, amphibians, and fish, the amino acid sequence of this transmembrane domain is poorly conserved, suggesting an anchor-only function at the outer mitochondrial membrane, rather than any specific role in signal transduction (Additional file 1: Fig. S4A). Protein topology analysis showed that mitochondrial targeting and insertion of Synj2bp in the outer membrane requires three conserved basic amino acid residues within the six-residue C-terminus, which is located in the mitochondrial intermembrane space [19]. Most of the N-terminal portion of Synj2bp spanning amino acids 1–119, comprises a PDZ domain (amino acids 21–100) that is exposed to the cytosol [19, 47]. Based on an evolutionary analysis of these structural features, only higher animals appear to have an ortholog of Synj2bp (Additional file 1: Fig. S4B). This suggests that this protein is an evolutionary innovation that allowed a PDZ domain to be anchored on the mitochondrial surface.

A recent study reported that Synj2bp binds RNA [46]; however, this association is puzzling due to the lack of any obvious RNA-binding domain in this protein. All other studies have reported interaction between Synj2bp and other proteins through its PDZ domain, which is known to coordinate protein–protein interactions [48, 49]. Among the binding partners of Synj2bp are the RNA-binding protein Synj2 [44] and the ER protein Rrbp1 [1, 13, 16]. Synj2bp association with these proteins confers distinct, critical functions to the mitochondrion. Specifically, the binding of Synj2a allows the mitochondria to retain specific gene transcripts, such as that of Pink1, to ensure a stable supply of the respective proteins [44]. Synj2bp binding to Rrbp1 allows the mitochondria to establish contacts with the ER [13, 16]. It should be noted, however, that all the functional studies on Synj2bp published so far were performed in vitro using cultured cells, where metabolism and cellular responses can differ from in vivo primary cells. To our knowledge, the work presented here is the first to explore Synj2bp expression and function in vivo*.* Its findings support studies that have assigned this protein a role in regulating mitochondria-rough-ER contacts in cultured cells [13, 16]. Such findings do not support, but neither do they rule out, a role for Synj2bp in binding RNA directly [46] or indirectly, via Synj2 [44]. In this regard, it should be noted that in the mouse liver, Synj2 expression is lower than 2 transcripts per million [1], a level so low that it should be questioned until properly validated. Therefore, in the liver, the function of Synj2bp in mRNA localization on the mitochondrial surface, if any, is probably mediated by its association with RNA-binding proteins that remain to be discovered.

Our study showed that Synj2bp silencing increases the expression of Carbamoyl phosphate synthetase 1 (Cps1; Fig. 6B and Additional file 1: Fig. S3G), the rate-limiting enzyme of the urea cycle, suggesting a possible upregulation of this pathway. The role of wrappER-mitochondria contacts on hepatic nitrogen metabolism remains to be addressed, but this aspect is of noteworthy because the transition from NAFLD to NASH also involves a loss of control of ammonia detoxification by the urea cycle [50–52].

In this study, we showed that, in the mouse liver, 75% knockdown of Synj2bp protein expression causes a major alteration in hepatic lipid flux. This result phenocopies liver in which the expression of Rrbp1, a protein that interacts with Synj2bp [16], was knocked down by 75% [1]. We also showed that Synj2bp is endogenously overexpressed in steatotic *ob/ob* mouse livers, where the extent of mitochondrial surface area covered by wrappER is chronically very large. Therefore, it appears most likely that Synj2bp is part of the pathway that, through the tethering of mitochondria to the wrappER, regulates hepatic lipid flux.

## Methods

### Animals

Adult male C57BL/6 N mice (8 weeks old) were purchased from Charles River, while adult male B6.Cg-*Lepob*/J homozygous mice (Strain No: 000632; 10 weeks old) were purchased from Jackson Laboratory. The mice were housed in a pathogen-free animal facility under a 12 h light/dark cycle at constant temperature and humidity and fed standard rodent chow and water ad libitum. All experiments were conducted with male mice 9–12 weeks old. For fasting/refeeding studies, animals were either fasted for 14 h overnight and sacrificed in the morning or fasted for 12 h overnight, and then, in the morning, they were fed with standard rodent chow ad libitum for 3 h. Animals were anesthetized with 2% isoflurane and sacrificed through cervical dislocation, then the livers were immediately excised. For liver cryo-fixation, animals were anesthetized by intraperitoneal injection with ketamine; 3–5 tissue biopsies were collected using the Rapid Transfer System (Leica) and vitrified by high-pressure-freezing (Leica EM PACT2). Mice were then sacrificed by cervical dislocation. All experiments involving animals were approved by the animal protection committee of Laval University (CPAUL) and performed in accordance with its guidelines for animal welfare.

### Immunoblot analysis

Total protein concentration was determined using the Bradford assay (Coomassie Protein Assay Reagent; Thermo Fisher Scientific). Protein samples were analyzed by SDS-PAGE using the following types of precast gels according to the manufacturer’s instructions: NuPAGE 3–8% Tris–Acetate Protein gels, Bolt 8% Bis–Tris Plus gels, Bolt 12% Bis–Tris Plus gels, Bolt 4–12% Bis–Tris Plus gels (Thermo Fisher Scientific). For western blotting, proteins were generally transferred for 60 min at 100 V to a PVDF membrane (Immobilon, Millipore; 0.45 μm pore size) in transfer buffer (20% methanol; 320 mM glycine, 20 mM Tris-base pH 8.4). For blots that were analyzed with anti-Synj2bp and anti-ApoB antibodies, proteins were transferred for 25 min or 90 min at 100 V, respectively. Blocking was performed for 60 min at room temperature with 7.5% nonfat milk or 8% bovine serum albumin (BSA; Sigma-Aldrich) in Tris-buffered saline, 0.1% Tween-20. Primary antibodies were incubated overnight at 4 °C in 5% nonfat milk or 5% BSA. The primary antibodies used were the following: β-actin (Cell Signaling Technology #4970; 1:10,000), AIF (Cell Signaling Technology #5318; 1:3,000), ApoB (rabbit polyclonal Abcam #ab20737 [36]; 1:2,000), ApoE (Abcam #Ab20874 [53, 54]; 1:3,000), Calnexin (StressMarq Biosciences #SPC-127; 1:15,000–20,000), Calreticulin (Cell Signaling Technology #12238; 1:120,000–1:150,000), Erp44 (Cell Signaling Technology #3798; 1:1,000), MnSOD (Enzo Life Sciences #ADI-SOD-110-D; 1:7000–1:20,000), Pdh (Cell Signaling Technology #3205, 1:2000), phospho-Pdh (Cell Signaling Technology #31866, 1:2000), phospho-S6 (Cell Signaling Technology #5364, 1:2,000), Synj2bp (Sigma-Aldrich #HPA000866; 1:1000–1:2000). The HRP-conjugated secondary antibodies used were the following: anti-Mouse IgG (Jackson ImmunoResearch #115-035-062; 1:5,000–10,000), anti-rabbit IgG (GE Healthcare #NA934; 1:5000–10,000). Protein bands were detected by chemiluminescence using the SuperSignal ELISA Femto substrate (Thermo Fisher Scientific) and the VersaDoc 3000 CCD imaging system (Bio-Rad Laboratories).

### Lambda protein phosphatase assay

15 μg of mouse liver heavy membranes was incubated with 0.2 μl of Lambda protein phosphatase according to the manufacturer’s instructions (New England Biolabs #P0753S).

### Plasma triglycerides (TAG) and cholesterol analysis

Mice were fasted for 6 h, and then 60 μl of blood was collected from the caudal vein in heparinized capillary tubes and immediately centrifuged at 2,100* g* for 12 min to prepare plasma fractions. Plasma was flash-frozen in dry ice and stored at − 80 °C until needed. Plasma TAG and total cholesterol measurements were performed using commercial colorimetric assay kits according to the manufacturer’s instructions (Pointe Scientific Inc.).

### Liver triglycerides (TAG) and cholesterol analysis

For neutral lipids analysis, 0.2 g of liver tissue was collected from fed mice. The sample was resuspended in 2 ml ddH_2_O supplemented with 1 × Protease Inhibitor Cocktail, and then mechanically homogenized at 1600 rpm for 1 min and 30 s in a 2 ml glass-Teflon Dounce homogenizer (Wheaton). 100 μl of homogenate was transferred to a 1.5 ml Eppendorf and brought under a chemical hood. Here, 125 μl of cold chloroform and 250 μl of cold methanol were added to the solution, then the sample was vortexed prior to a 5 min incubation on ice. Next, 125 μl of cold chloroform and 125 μl of cold ddH_2_O were mixed with the sample and the solution was vortexed. After centrifuging the sample for 5 min at 3000* g* at 4 °C, 150 μl of the lower phase was collected, mixed with 150 μl of chloroform containing 4% Tritonx100, vortexed, and dried for 15 min at 45 °C using a SpeedVac. The lipidic pellet was resuspended in 300 μl of methanol, vortexed, and sonicated to achieve complete resuspension of the lipids. TAG and total cholesterol were then measured using commercial colorimetric assay kits according to the manufacturer’s instructions (Pointe Scientific Inc.).

### Preparation of fractions enriched in wrappER-associated mitochondria (WAM) from mouse livers

WAM-enriched fractions were isolated as described [42]. Briefly, the mouse liver was quickly excided, washed three times with Buffer A (BA; 10 mM Tris–HCl, 1 mM MgCl_2_, 0.1 mM EGTA, pH 7.4) supplemented with 250 mM Sucrose (B10), minced in small pieces, and resuspended in 5 ml B10 supplemented with 1 × Protease Inhibitor Cocktail (PIC; Thermo Fisher Scientific, #78429). The sample was mechanically homogenized with 16 strokes in a glass-Teflon Dounce homogenizer (Wheaton, #358034), brought to 12 ml volume with B10 supplemented with PIC, and poured into two 30 ml glass centrifuge tubes. The homogenate was spun three times at 400* g* for 10 min at 4 °C, and at the end of the centrifugation, the supernatants were collected and mixed to obtain the mouse liver whole lysate (WL). 7 ml of WL were carefully layered on top of 7 ml of a 27% w/w sucrose solution (27% sucrose in BA) inside a 50 ml Falcon tube and spun at 2000* g* for 20 min at 4 °C; the pellet thus obtained represents the WAM-enriched fraction.

#### Heavy Membranes (HM) fractions preparation

1 ml of WL was transferred into a 1.5 ml Eppendorf and spun at 7000* g* for 10 min at 4 °C; the supernatant was discarded, and the obtained pellet (HM fraction) was resuspended in B10 supplemented with PIC.

### Liver EM analysis

Liver biopsies were cryo-fixed using the Leica high-pressure freezer EM PACT2 (Leica Microsystems). After that, the Leica Automatic Freeze Substitution (AFS) was used to freeze-substitute samples. Acetone containing 1% OsO_4_ and 0.1% uranyl acetate was used as substitution medium. The procedure started at – 90 °C for 10 h and then the temperature was raised to – 60 °C at the rate of 5 °C/h; the incubation at – 60 °C lasted for 12 h, then the samples were warmed up to – 30 °C at the rate of 5 °C/h. After 8 h at – 30 °C, the temperature was raised to 0 °C at the rate of 1 °C/min. At this point, the substitution medium was replaced with pure acetone. Samples were embedded in an Araldite/Epon/Dodecenylsuccinic anhydride (DDSA) and 2,4,6-tris (dimethylaminomethyl) phenol (DMP30) mixture [araldite/epon stock, epoxy 41% (wt/wt), durcupan Araldite casting resin M (ACM) 54% (wt/wt), dibutylphthalate 5% (wt/wt); araldite/epon complete formulation, araldite/epon stock 49% (wt/wt), hardener DDSA 49% (wt/wt), and accelerator DMP-30 2% (wt/wt)]. The procedure was performed stepwise: 33% resin in water-free acetone for 3 h at 4 °C, 66% resin in water-free acetone for 3 h at 4 °C, 100% resin overnight at RT, and a 100% resin change before polymerization. All samples were polymerized at 58 °C for at least 48 h. Samples were cut at 50 nm and put on single-slot copper grids using a Leica Ultramicrotome. After counterstaining with lead citrate, samples were imaged on a Tecnai-12 by Philips with a Megaview camera using the Analysis software.

### EM morphometric analysis

All the morphometric studies shown in the manuscript were conducted on high-quality TEM images of mouse liver cryo-fixed biopsies. Measures collection was performed using ImageJ (NIH). Data were analyzed and plotted using Excel (v.16.25; Microsoft), Prism (v.9; GraphPad), and https://www.estimationstats.com [24].

### Lipoprotein imaging by negative staining and immunogold EM analysis

Livers of mice at 3 h postprandial were excised and WAM-enriched fractions were freshly prepared as described in [42]. The WAM-containing pellet was resuspended in 600 μl of TBS (150 mM NaCl, 10 mM Tris–HCl pH 7.6) supplemented with 1 × PIC (Thermo Fisher Scientific). Membranes (containing approx. 1 mg of proteins) were permeabilized by adding 600 μl of TBS 0.1% Triton X-100, followed by incubation at 4 °C for 30 min and deep freeze-and-thaw. The permeabilized WAM-enriched fractions were transferred to a 4 ml ultracentrifuge tube containing 1.8 ml of 0.5 × TBS and centrifuged for 1 h at 400,000* g* in an SW60-Ti rotor (Beckman Coulter). Floated lipoproteins were recovered by skimming the top 100 μl of the supernatant and then 2.5 μl was placed on glow-discharged carbon-coated 300 mesh copper grids (SPI Supplies). After 5 min incubation at RT, the grids were blocked for 10 min with 50 μl of PBSB (PBS, 0.5% BSA-c; Electron Microscopy Sciences), followed by incubation either with anti-ApoE rabbit polyclonal antibody diluted 1:50 in PBSB for 30 min at RT (Abcam #ab52607, lot #GR103096-3) [36] or with anti-ApoB rabbit polyclonal antibody (diluted 1:50 in PBSB; Abcam #ab20737 [36], lot #GR3176056-6). Grids were then washed 3 times at RT with PBSB (2 min per wash) and incubated at RT for 30 min with gold-conjugated (10 nm for ApoE, 15 nm for ApoB) secondary antibody (diluted 1:100 in PBSB; Goat-anti-Rabbit lgG (H&L), Electron Microscopy Sciences #25109). After three washes in PBSB, the grids were stained with 1% uranyl formate according to well-established EM protocols optimized for lipoproteins studies [55]. Images were acquired with a Tecnai-12 TEM microscope (Philips).

### Quantitative real-time PCR

Livers of six fasted and fed mice (3 h postprandial) were excised, and a small piece of tissue was flash-frozen in liquid N_2_. Frozen tissues were homogenized in Qiazol buffer (Qiagen) and total RNA was extracted using the miRNeasy micro kit on-column DNase (Qiagen) treatment following the manufacturer’s instructions. The quantity of total RNA was measured using the NanoDrop ND-1000 Spectrophotometer (NanoDrop Technologies) and total RNA quality was assayed on an Agilent BioAnalyzer 2100 (Agilent Technologies). First-strand cDNA synthesis was accomplished using 4 μg of isolated RNA in a reaction containing 200 U of Superscript IV Rnase H-RT (Invitrogen Life Technologies), 300 ng of oligo-dT_18_, 50 ng of random hexamers, 50 mM Tris–HCl pH 8.3, 75 mM KCl, 3 mM MgCl_2_, 500 μM deoxynucleotides triphosphate, 5 mM dithiothreitol, and 40 U of Protector RNase inhibitor (Roche Diagnostics) in a final volume of 50 μl. The reaction was incubated at 25 °C for 10 min, then at 50 °C for 20 min, and inactivated at 80 °C for 10 min. PCR purification kit (Qiagen) was used to purify cDNA. Oligoprimer pairs were designed by GeneTool 2.0 software (Biotools Inc, Edmonton, AB, CA) and their specificity was verified by blast in the GenBank database. The synthesis was performed by IDT (Integrated DNA Technology). cDNA corresponding to 20 ng of total RNA was used to perform fluorescent-based Realtime PCR quantification using the LightCycler 480 (Roche Diagnostics). Reagent LightCycler 480 SYBRGreen I Master (Roche Diagnostics) was used as described by the manufacturer. The conditions for PCR reactions were: 45 cycles, denaturation at 98 °C for 10 s, annealing at 62 °C for 10 s, elongation at 72 °C for 14 s, and reading for 5 s. A melting curve was performed to assess non-specific signals. Relative quantity was calculated using second derivative method and by applying the delta Ct method [56]. Normalization was performed using as reference transcripts of genes with stable expression levels from embryonic life through adulthood in various tissues [57]: ATP synthase H + transporting mitochondrial F1 complex O subunit (*Atp5o*), hypoxanthine guanine phosphoribosyl transferase 1 (*Hprt1*) and glyceraldehyde-3-phosphate dehydrogenase (*Gapdh*). Quantitative Real-Time PCR measurements were performed by the CHU de Québec Research Center (CHUL) Gene Expression Platform, Quebec, Canada and were compliant with MIQE guidelines [58].

## Sequence primers and gene description

**Table:** 

Gene symbol	Description	GenBank	Size (bp)	Primer sequence 5′ → 3′ S/AS
*Synj2bp*	*Mus musculus* Synaptojanin 2 binding protein (Synj2bp), 2 coding RNA transcripts	NM_025292/NM_001309814	130	TCGGTGGGACAGATCAACAGTA/TGGCCATTTACCGAGAGGATC
*Atp5o*	*Mus musculus* ATP synthase, H + transporting, mitochondrial F1 complex, O subunit (Atp5o)	NM_138597	142	GCTATGCAACCGCCCTGTACTCTG/ACGGTGCGCTTGATGTAGGGATTC
*Hprt1*	*Mus musculus* hypoxanthine guanine phosphoribosyl transferase 1 (Hprt1)	NM_013556	106	CAGGACTGAAAGACTTGCTCGAGAT/CAGCAGGTCAGCAAAGAACTTATAGC
*Gapdh*	*Mus musculus* glyceraldehyde-3-phosphate dehydrogenase (Gapdh)	NM_008084	194	GGCTGCCCAGAACATCATCCCT/ATGCCTGCTTCACCACCTTCTTG
gDNA	*Mus musculus* chromosome 3 genomic contig, strain C57BL/6 J (Hsd3b1 intron)	NT_039239	209	CACCCCTTAAGAGACCCATGTT/CCCTGCAGAGACCTTAGAAAAC

### Protein mass spectrometry analysis

Six liver WAM-enriched fractions from AAV8-shScr- and AAV8-shSynj2bp-injected mice were used as samples. Proteins were precipitated with acetone, then pellets were resuspended in Buffer-1 (50 mM ammonium bicarbonate, 1% sodium deoxycholate, pH 8), and quantified using the Micro-Bradford assay (Bio-Rad). 10 µg of proteins were reduced with 0.2 mM DTT for 30 min at 37 °C and alkylated with 0.8 mM iodoacetamide for 30 min at 37 °C. Samples were then incubated with trypsin (trypsin:protein; 1:50) at 37 °C overnight. The reaction was stopped by adding 1% TFA, 0.5% acetic acid, and 0.5% acetonitrile then centrifuged for 5 min at 13,000 rpm. The peptides were desalted using C18 stagetip. 1 µg of each sample was analyzed by nano LC–MS/MS using a Dionex UltiMate 3000 nanoRSLC chromatography system (Thermo Fisher Scientific) connected to an Orbitrap Fusion mass spectrometer (Thermo Fisher Scientific) equipped with a nanoelectrospray ion source. Peptides were trapped at 20 μl/min in loading solvent (2% acetonitrile, 0.05% TFA) on a 5 mm × 300 μm C18 pepmap cartridge pre-column (Thermo Fisher Scientific) for 5 min. Then, the pre-column was switched online with Pepmap Acclaim column (ThermoFisher) 50 cm × 75 µm internal diameter separation column and the peptides were eluted with a linear gradient from 5 to 40% solvent B (A: 0.1% formic acid, B: 80% acetonitrile, 0.1% formic acid) in 90 min, at 300 nl/min for a total run time of 120 min. Mass spectra were acquired using a data-dependent acquisition mode using Thermo XCalibur software version 4.1.50. Full scan mass spectra (350–1800 m/z) were acquired in the orbitrap using an AGC target of 4e5, a maximum injection time of 50 ms, and a resolution of 120,000. Internal calibration using lock mass on the m/z 445.12003 siloxane ion was used. Each MS scan was followed by the acquisition of fragmentation MS/MS spectra of the most intense ions for a total cycle time of 3 s (top speed mode). The selected ions were isolated using the quadrupole analyser in a window of 1.6 m/z and fragmented by Higher energy Collision-induced Dissociation (HCD) with 35% of collision energy. The resulting fragments were detected by the linear ion trap in rapid scan rate with an AGC target of 1e4 and a maximum injection time of 50 ms. Dynamic exclusion of previously fragmented peptides was set for a period of 30 s and a tolerance of 10 ppm.

### Proteomics analysis

Spectra were searched against the Uniprot Ref *Mus musculus* database (July 2020 release/63,738 entries) using the Andromeda module of MaxQuant software v. 1.6.10.43 [59]. Trypsin/P enzyme parameter was selected with two possible missed cleavages. Carbamidomethylation of cysteins was set as a fixed modification while methionine oxidation and protein N-terminal acetylation were set as variable modifications. Mass search tolerances were 5 ppm and 0.5 Da for MS and MS/MS respectively. For protein validation, a maximum False Discovery Rate of 1% at peptide and protein level was used based on a target/decoy search. MaxQuant was also used for Label Free Quantification. The ‘match between runs’ option was used with a 20 min value as alignment time window and 0.7 min as match time window. Only unique and razor peptides were used for quantification. Normalization (LFQ intensities) was performed by MaxQuant.

#### Criteria for protein identification

RStudio 1.2.5019 was used for data post-processing. Missing protein intensity values were replaced by a noise value corresponding to 1% percentile of the normalized value for each condition. A protein was considered quantifiable only if (1) the intensity values for the protein under investigation were present in all replicates of one of the two compared conditions, and (2) two or more peptides of the protein were identified. Protein digestion, mass spectrometry analyses, and protein identification were performed by the Proteomics Platform of the CHU de Quebec-Université Laval Research Center, Quebec City, Canada.

#### Software analyses

The identified proteins were plotted as a volcano graph using Prism (v.9, GraphPad Software). Significantly upregulated proteins were considered those with *z* score > 1.96 and *p* value < 0.05. In contrast, significantly downregulated proteins had *z* score < − 1.96 and *p* value < 0.05 [60]. g:Profiler [61] was used for functional enrichment analysis. A list of upregulated proteins with a *q* value (Benjamin-Hochberg correction for multiple testing) < 0.05 was chosen as input, and g:Profiler was set up by selecting Benjamini–Hochberg FDR as test correction and Reactome for biological pathway analysis. Pathways represented by a single protein were excluded from the results.

### AAV8-shRNA design and in vivo delivery

An adeno-associated virus serotype 8 (AAV8) encoding a short hairpin RNA (shRNA) construct targeting mouse *Synj2bp* (Genebank RefSeq NM_025292; targeting sequence: CATCTACGTCAGCCGTATCAA; shRNA sequence: 5’-CCGGCATCTACGTCAGCCGTATCAA-CTCGAG-TTGATACGGCTGACGTAGATG-TTTTTG-3’) driven by the U6 promoter was purchased from Vector Biolabs; this vector (AAV8-GFP-U6-mSynj2bp-shRNA) also expressed eGFP driven by the CMV promoter and yielded ~ 90% knockdown of Synj2bp expression in Hepa1.6 cells (Vector Biolabs). The control vector (AAV8-GFP-U6-scrmb-shRNA) encoded a short hairpin with a scrambled sequence (5’- CAACAAGATGAAGAGCACCAA-3’; Vector Biolabs). AAV8 particles were purified by two rounds of CsCl gradient purification, desalted, and titered (titer: > 2 × 10^13^ GC/ml). AAV8 particles were diluted in sterile PBS and wild-type C57BL/6N mice (9–12 weeks old) were injected with either control vector (AAV8-shScr) or AAV8-GFP-U6-mSynj2bp-shRNA (AAV8-shSynj2bp) via tail vein injection (1.8 × 10^11^ genome copies per mouse) with BD Ultra-Fine Insulin Syringes. Experiments began 21 days after injection.

### Figures preparation

Histograms, dot plots, and volcano plots were generated with Prism (v.9, GraphPad Software), estimation charts with https://www.estimationstats.com/, and pie charts with https://www.onlinecharttool.com. Figure 4A was partly created using Servier Medical Art (https://smart.servier.com/); Fig. 6B was created using the circlize package in RStudio (2022.07.1); Additional file 1: Fig. S4B was created based on the Ensembl (http://useast.ensembl.org/) *Synj2bp* phylogenetic tree. Affinity Designer (RRID:SCR_016952) was used to edit and arrange the figures.

### Data reporting

No statistical methods were used to predetermine sample size. The experiments were randomized, and the investigators were not blinded to allocation during experiments and outcome assessment. See Additional file 3 for the original, uncropped immunoblots.

### Quantification and statistical analysis

Unpaired *t* test was used for comparisons involving two groups. For data sets from two groups that did not fulfill the D'Agostino and Pearson omnibus normality test (α = 0.05), differences were assessed using a nonparametric two-tailed Mann–Whitney test with 95% confidence.

*P* values were calculated using Prism (v. 9, GraphPad Software) as specified in the figure legends. *P* values of less than 0.05 were considered statistically significant: n.s. *p* > 0.05, *p < 0.05, **p < 0.01, ***p < 0.001, ****p < 0.0001. Sample sizes for each experiment are displayed in the figures. Histograms were compiled to include mean ± SEM (standard error of the mean) unless otherwise indicated. For estimation statistics based on confidence intervals [24, 25], raw data were directly introduced in https://www.estimationstats.com/, downloaded the results, and plotted them as a Gardner-Altman estimation chart. In these graphs, the two different groups are plotted on the left side, whereas on the right is the bootstrap sampling distribution. The mean difference between the groups is depicted as the dot within the vertical error bar that represents the 95% confidence interval [24].

## Supplementary Information

**Additional file 1.** **Fig. S1.** Synj2bp silencing in the mouse liver alters mitochondrial morphology. **Fig. S2.** The wrappER contains ApoB-containing lipoparticles. **Fig. S3.** Synj2bp silencing in the mouse liver upregulates mitochondria fatty acid respiration capacity while down-regulating ApoE and Mups expression at the WAM. **Fig. S4.** Synj2bp evolutionary analysis.

**Additional file 2. Table S1.** Comparative quantitative proteomic analysis of WAM-enriched fractions isolated from sh-Scrambled and sh-Synj2bp mouse livers.

**Additional file 3.** Original, uncropped immunoblots.

## Data Availability

All data are available in the manuscript or in the supplementary materials. All mass spectrometry data (raw files and MaxQuant search result files) are publicly available on ProteomeXchange repository (www.proteomexchange.org) with the identifier PXD036681.

## Abbreviations

WAM: WrappER-associated mitochondria
VLDL: Very-low-density lipoprotein
Synj2bp: Synaptojanin-2-binding protein
FA: Fatty acid
MAM: Mitochondria-associated ER membrane
sh-scr: Sh-scrambled
