# Supplementary material for: Expression of Synj2bp in mouse liver regulates the extent of wrappER-mitochondria contact to maintain hepatic lipid homeostasis
Source: Biol Direct. 2022 Dec 1;17:37. doi: 10.1186/s13062-022-00344-8 (PMC9717519; doi:10.1186/s13062-022-00344-8)
Supplement: Supplementary file 1 — Additional file 1. Fig. S1. Synj2bp silencing in the mouse liver alters mitochondrial morphology. Fig. S2. The wrappER contains ApoB-containing lipoparticles. Fig. S3. Synj2bp silencing in the mouse liver upregulates mitochondria fatty acid respiration capacity while down-regulating ApoE and Mups expression at the WAM. Fig. S4. Synj2bp evolutionary analysis. [file 13062_2022_344_MOESM1_ESM.pdf]

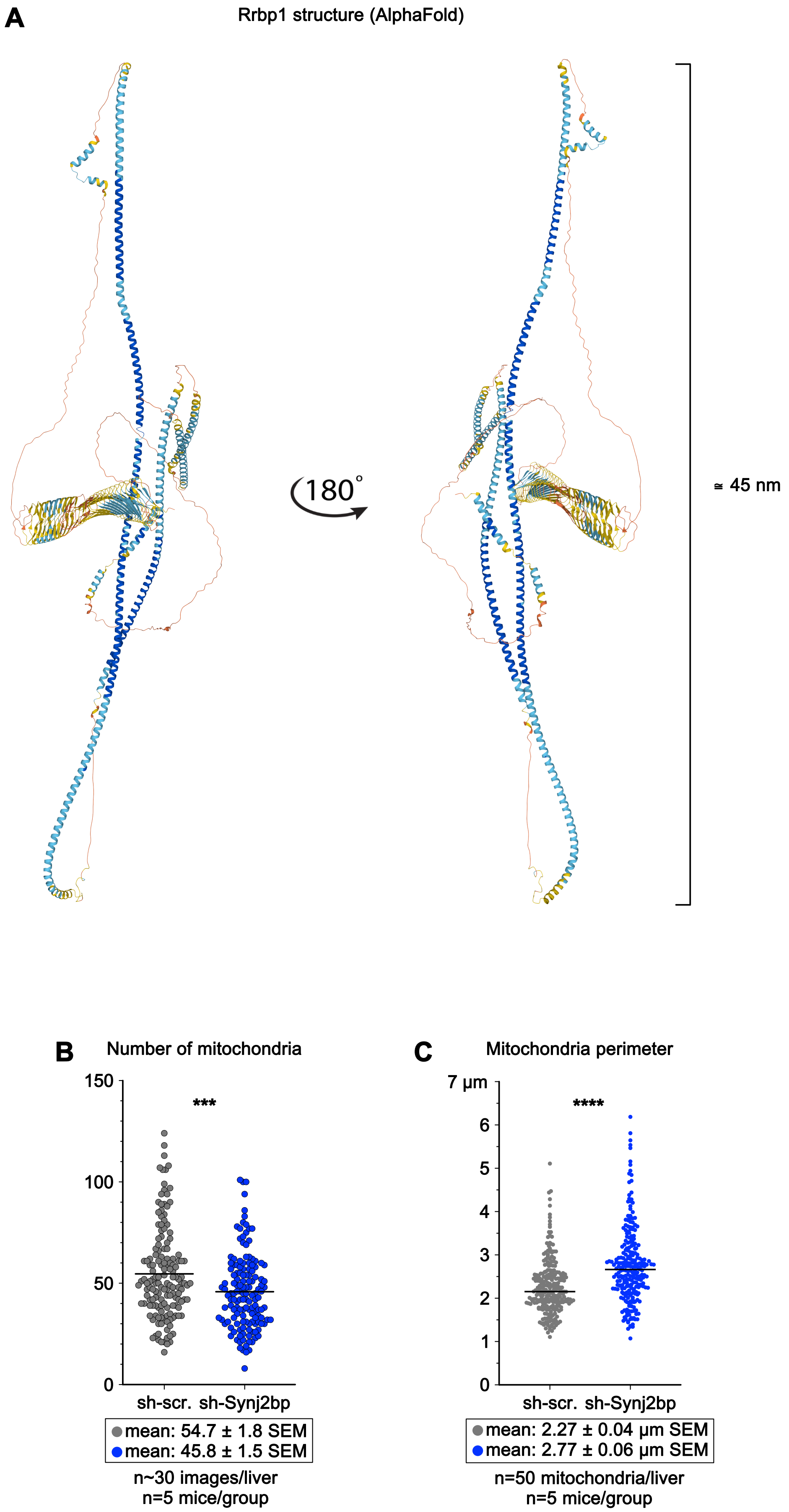
A
Rrbp1 structure (AlphaFold)
180°
≅ 45 nm
B
Number of mitochondria
150
100
50
0
***
sh-scr.
sh-Synj2bp
mean: 54.7 ± 1.8 SEM
mean: 45.8 ± 1.5 SEM
n~30 images/liver
n=5 mice/group
C
Mitochondria perimeter
7 µm
6
5
4
3
2
1
0
****
sh-scr.
sh-Synj2bp
mean: 2.27 ± 0.04 µm SEM
mean: 2.77 ± 0.06 µm SEM
n=50 mitochondria/liver
n=5 mice/group

**Fig. S1 Synj2bp silencing in the mouse liver alters mitochondrial morphology.**

**A** AlphaFold-predicted 3D structure of mouse Rrbp1, which regulates wrappER-mitochondria contact distance [1]. **B**,**C** Quantitative EM morphometric analyses of the number and size (perimeter) of mitochondria present in control and Synj2bp-silenced livers. In **B**, each dot represents the number of mitochondria counted in a single low-magnification EM image of livers collected from mice injected with control and AAV8-shSynj2bp vectors. In **C**, each dot represents the length of the perimeter of the mitochondria imaged in high-magnification EM images. This study shows that Synj2bp-silenced livers have fewer but larger mitochondria (mice at 3 h postprandial). The Student's *t* test was used to calculate *p* values.

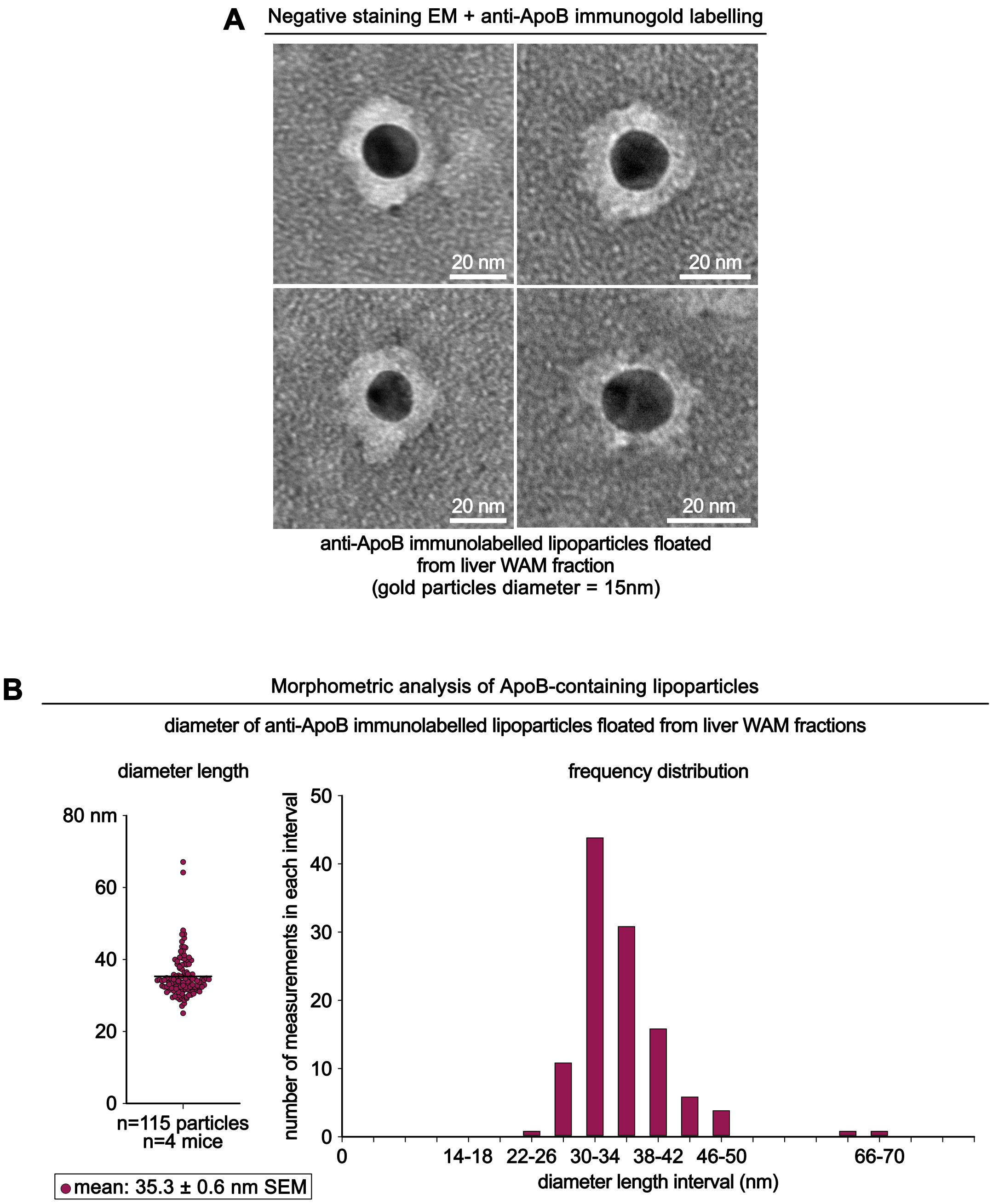
A
Negative staining EM + anti-ApoB immunogold labelling
20 nm
20 nm
20 nm
20 nm
anti-ApoB immunolabelled lipoparticles floated
from liver WAM fraction
(gold particles diameter = 15nm)
B
Morphometric analysis of ApoB-containing lipoparticles
diameter of anti-ApoB immunolabelled lipoparticles floated from liver WAM fractions
diameter length
80 nm
60
40
20
0
n=115 particles
n=4 mice
mean: 35.3 ± 0.6 nm SEM
frequency distribution
number of measurements in each interval
50
40
30
20
10
0
0
14-18
22-26
30-34
38-42
46-50
66-70
diameter length interval (nm)

**Fig. S2 The wrappER contains ApoB-containing lipoparticles.**

**A,B** Negative staining EM coupled to immunogold labeling of anti-ApoB lipoparticles floated from mouse liver WAM-enriched fractions. **B** Size of ApoB-containing lipoparticles isolated from WAM fractions. The value of this parameter is expressed in nm and refers to the diameter of the lipoparticle. The right graph shows the frequency distribution of lipoparticle sizes. Gold particles diameter = 15 nm.
WAM: wrappER-associated mitochondria.

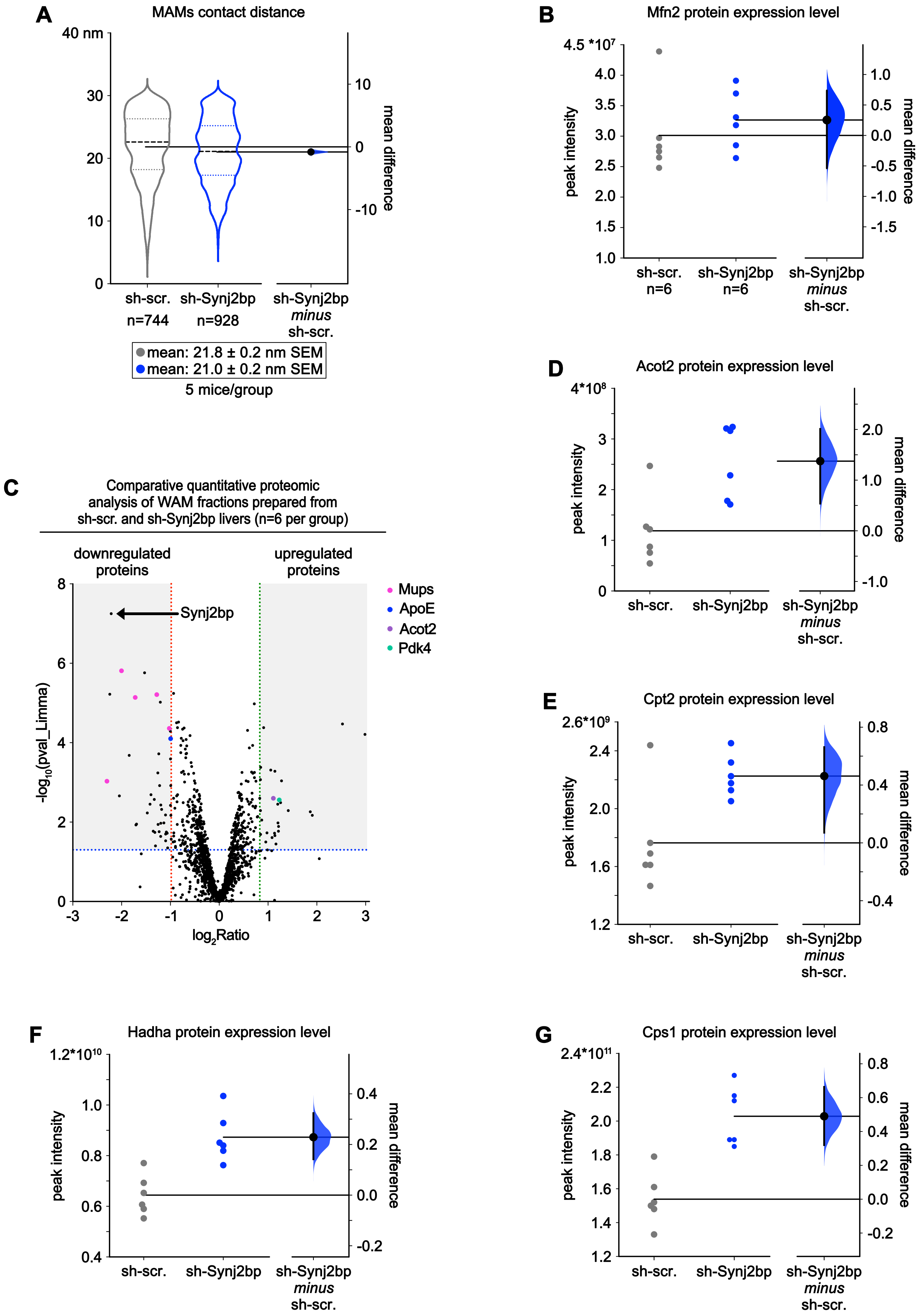

A
MAMs contact distance
40 nm
30
20
10
0
10
0
-10
mean difference
sh-scr.
n=744
sh-Synj2bp
n=928
sh-Synj2bp minus sh-scr.
mean: 21.8 ± 0.2 nm SEM
mean: 21.0 ± 0.2 nm SEM
5 mice/group
B
Mfn2 protein expression level
4.5 *10^7
peak intensity
mean difference
sh-scr. n=6
sh-Synj2bp n=6
sh-Synj2bp minus sh-scr.
C
Comparative quantitative proteomic analysis of WAM fractions prepared from sh-scr. and sh-Synj2bp livers (n=6 per group)
downregulated proteins
upregulated proteins
Synj2bp
Mups
ApoE
Acot2
Pdk4
-log10(pval_Limma)
log2Ratio
D
Acot2 protein expression level
4*10^8
peak intensity
mean difference
sh-scr.
sh-Synj2bp
sh-Synj2bp minus sh-scr.
E
Cpt2 protein expression level
2.6*10^9
peak intensity
mean difference
sh-scr.
sh-Synj2bp
sh-Synj2bp minus sh-scr.
F
Hadha protein expression level
1.2*10^10
peak intensity
mean difference
sh-scr.
sh-Synj2bp
sh-Synj2bp minus sh-scr.
G
Cps1 protein expression level
2.4*10^11
peak intensity
mean difference
sh-scr.
sh-Synj2bp
sh-Synj2bp minus sh-scr.

**Fig. S3 Synj2bp silencing in the mouse liver upregulates mitochondria fatty acid respiration capacity while down-regulating ApoE and Mups expression at the WAM.**

**A** Electron microscopy morphometric analyses of the MAMs contact distance in control and Synj2bp-silenced mouse livers. Data were processed by estimation statistics analysis **B** Estimation statistics analysis of the WAM proteomics data of the MAM tether Mfn2. **C** Comparative quantitative proteomic analysis. The volcano plot shows the proteins that are down- and up-regulated in WAM-enriched fractions isolated from Synj2bp-silenced livers compared to control AAV8-shScrambled injected mice. **D-G** Estimation statistics analysis of the WAM proteomics data related to the indicated proteins. **D-F** Enzymes that participate in mitochondrial FA respiration; their up-regulation indicates the activation of this catabolic process in Synj2bp-silenced livers. **G** Cps1 is the rate-limiting step of the urea cycle; its increased expression suggests a possible upregulation of this pathway.

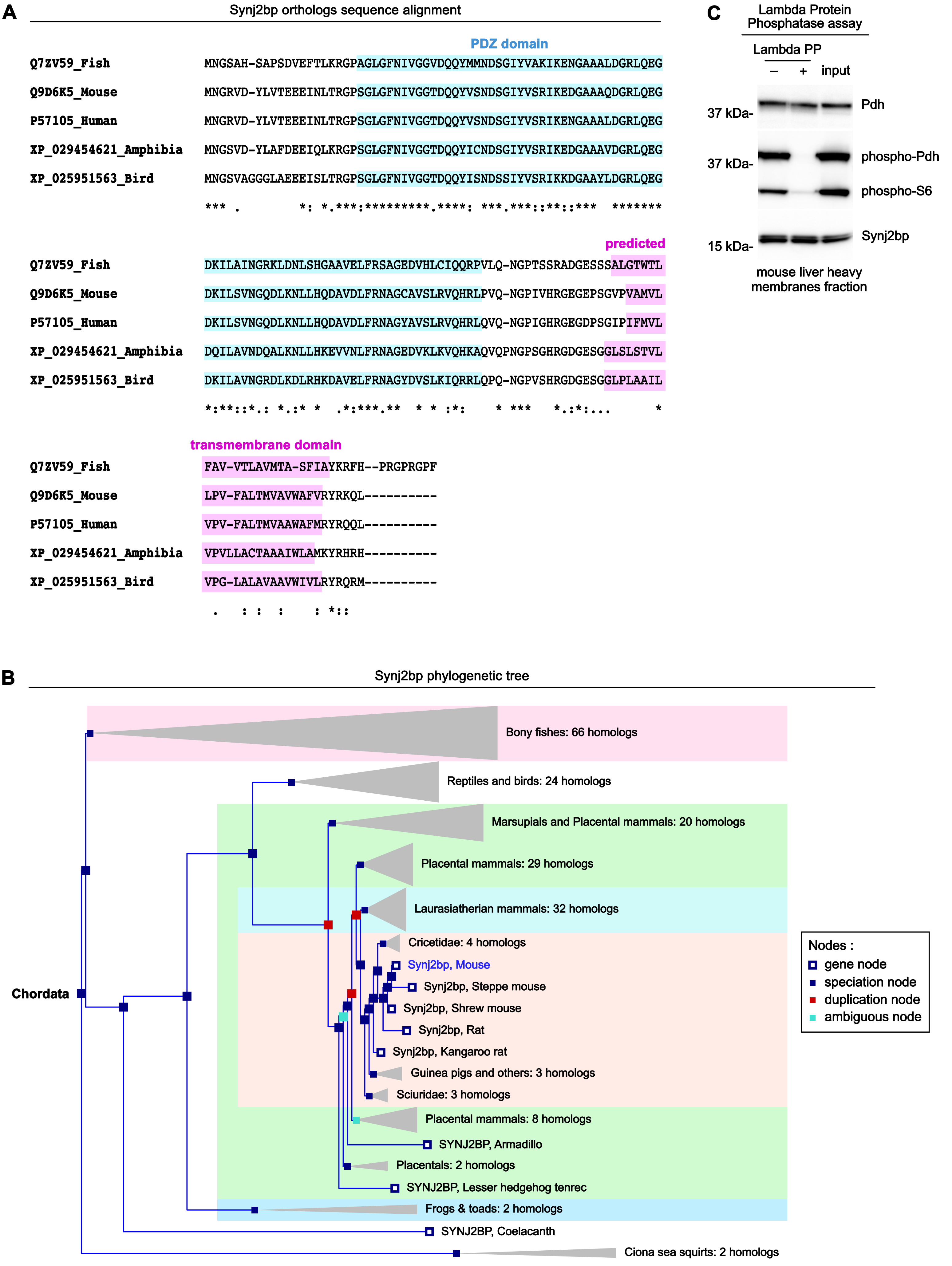
A
Synj2bp orthologs sequence alignment
PDZ domain
Q7ZV59_Fish MNGSAH-SAPSDVEFTLKRGPAGLGFNIVGGVDQQYMMNDSGIYVAKIKENGAAALDGRLQEG
Q9D6K5_Mouse MNGRVD-YLVTEEEINLTRGPSGLGFNIVGGTDQQYVSNDSGIYVSRIKEDGAAAQDGRLQEG
P57105_Human MNGRVD-YLVTEEEINLTRGPSGLGFNIVGGTDQQYVSNDSGIYVSRIKENGAAALDGRLQEG
XP_029454621_Amphibia MNGSVD-YLAFDEEIQLKRGPSGLGFNIVGGTDQQYICNDSGIYVSRIKEDGAAAVDGRLQEG
XP_025951563_Bird MNGSVAGGGLAEEEISLTRGPSGLGFNIVGGTDQQYISNDSSIYVSRIKKDGAAYLDGRLQEG
predicted
Q7ZV59_Fish DKILAINGRKLDNLSHGAAVELFRSAGEDVHLCIQQRPVLQ-NGPTSSRADGESSSALGTWTL
Q9D6K5_Mouse DKILSVNGQDLKNLLHQDAVDLFRNAGCAVSLRVQHRLPVQ-NGPIVHRGEGEPSGVPVAMVL
P57105_Human DKILSVNGQDLKNLLHQDAVDLFRNAGYAVSLRVQHRLQVQ-NGPIGHRGEGDPSGIPIFMVL
XP_029454621_Amphibia DQILAVNDQALKNLLHKEVVNLFRNAGEDVKLKVQHKAQVQPNGPSGHRGDGESGGLSLSTVL
XP_025951563_Bird DKILAVNGRDLKDLRHKDAVELFRNAGYDVSLKIQRRLQPQ-NGPVSHRGDGESGGLPLAAIL
transmembrane domain
Q7ZV59_Fish FAV-VTLAVMTA-SFIAYKRFH--PRGPRGPF
Q9D6K5_Mouse LPV-FALTMVAVWAFVRYRKQL----------
P57105_Human VPV-FALTMVAAWAFMRYRQQL----------
XP_029454621_Amphibia VPVLLACTAAAIWLAMKYRHRH----------
XP_025951563_Bird VPG-LALAVAAVWIVLRYRQRM----------
B
Synj2bp phylogenetic tree
Chordata
Bony fishes: 66 homologs
Reptiles and birds: 24 homologs
Marsupials and Placental mammals: 20 homologs
Placental mammals: 29 homologs
Laurasiatherian mammals: 32 homologs
Cricetidae: 4 homologs
Synj2bp, Mouse
Synj2bp, Steppe mouse
Synj2bp, Shrew mouse
Synj2bp, Rat
Synj2bp, Kangaroo rat
Guinea pigs and others: 3 homologs
Sciuridae: 3 homologs
Placental mammals: 8 homologs
SYNJ2BP, Armadillo
Placentals: 2 homologs
SYNJ2BP, Lesser hedgehog tenrec
Frogs & toads: 2 homologs
SYNJ2BP, Coelacanth
Ciona sea squirts: 2 homologs
Nodes :
gene node
speciation node
duplication node
ambiguous node
C
Lambda Protein Phosphatase assay
Lambda PP
– + input
37 kDa-
Pdh
37 kDa-
phospho-Pdh
phospho-S6
15 kDa-
Synj2bp
mouse liver heavy membranes fraction

**Fig. S4 Synj2bp evolutionary analysis.**

**A** Multiple sequence alignment of representative Synj2bp orthologs from the indicated vertebrate groups. **B** Schematic representation of the mouse Synj2bp phylogenetic tree. **C** Lambda Protein Phosphatase assay on mouse liver heavy membranes fraction. The image shows the immunoblot analysis done using a mix of four antibodies that recognize Synj2bp, Pdh, phosphorylated Pdh (Ser293), and phosphorylated S6 (Ser240/244), respectively. Note that phosphatase treatment abolishes the signal generated by phospho-specific antibodies, used here as positive controls. This data indicates that the two forms of Synj2bp are likely not the result of Ser/Thr/Tyr phosphorylation.
