# Supplementary material for: Expression of Synj2bp in mouse liver regulates the extent of wrappER-mitochondria contact to maintain hepatic lipid homeostasis
Source: Biol Direct. 2022 Dec 1;17:37. doi: 10.1186/s13062-022-00344-8 (PMC9717519; doi:10.1186/s13062-022-00344-8)

Additional file 3

Mouse liver WAM-enriched fractions (8 µg/lane)

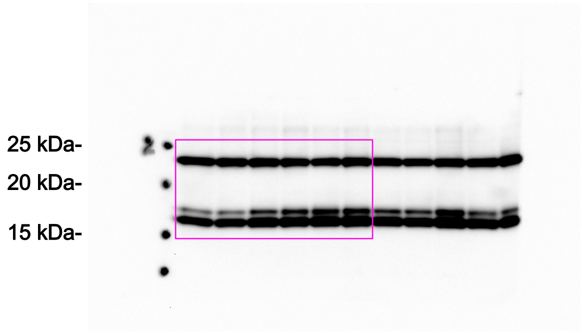

W.B. #1
anti-MnSOD 1:10,000
anti-Synj2bp 1:3,000

Mouse liver WAM-enriched fractions (10 µg/lane)

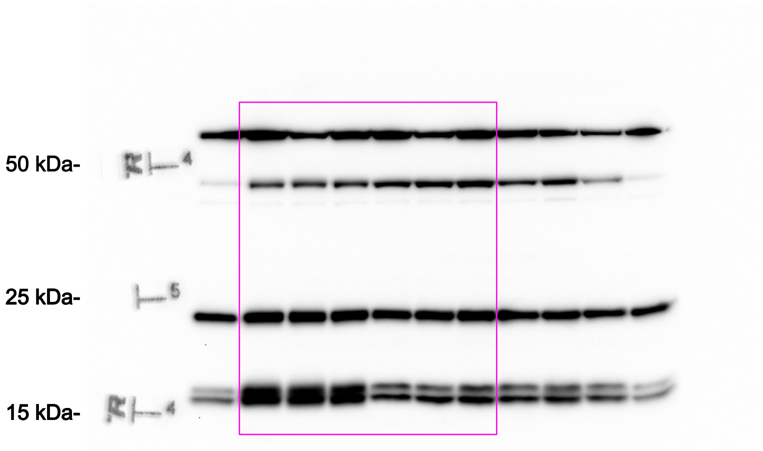

W.B. #2
anti-Aif 1:1,500
anti-Erp44 1:1,000
anti-MnSOD 1:14,000
anti-Synj2bp 1:1,000

Additional file 3

Mouse liver heavy membranes (40 µg/lane)

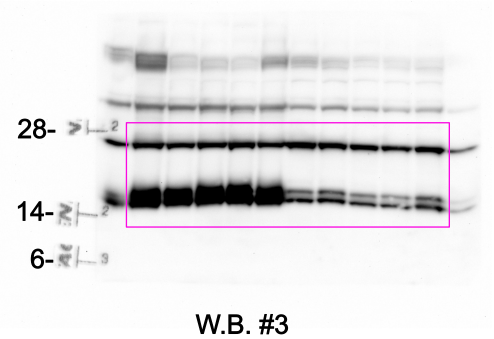

W.B. #3
anti-MnSod 1:7,000
anti-Synj2bp 1:750

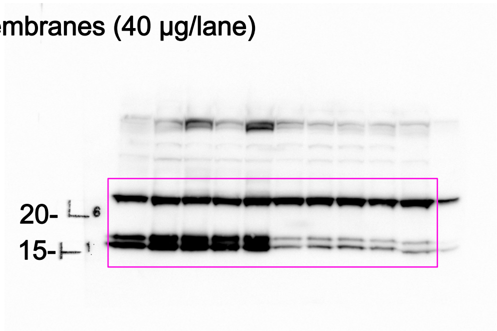

W.B. #4
anti-MnSod 1:7,000
anti-Synj2bp 1:750

Mouse liver lysate (20 µg/lane)

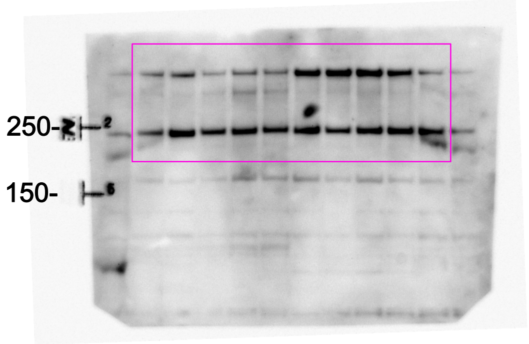

W.B. #5
anti-ApoB 1:2,000

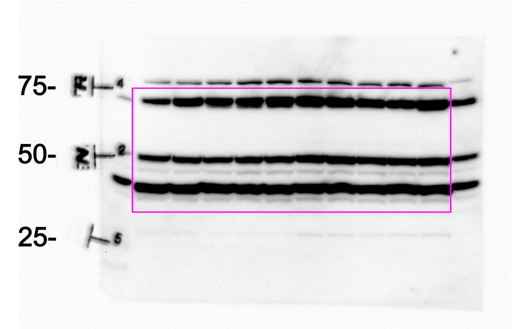

W.B. #6
anti-Calnexin 1:18,000
anti-Calreticulin 1:120,000
anti-Actin 1:15,000

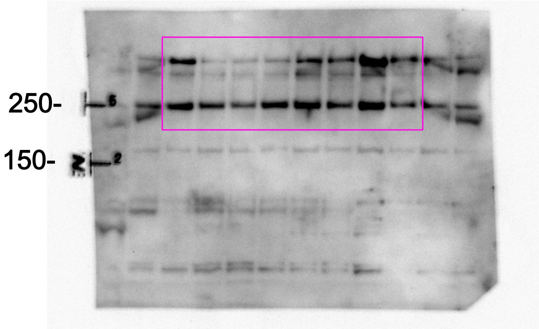

W.B. #7
anti-ApoB 1:2,000

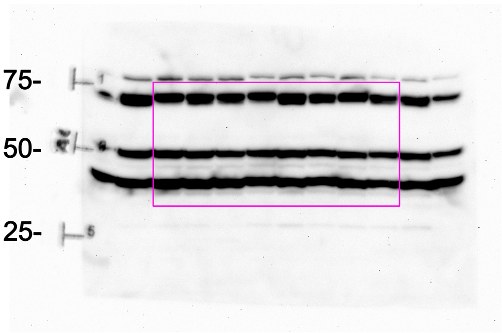

W.B. #8
anti-Calnexin 1:18,000
anti-Calreticulin 1:120,000
anti-Actin 1:15,000

Additional file 3

Mouse liver heavy membranes (20 µg/lane)

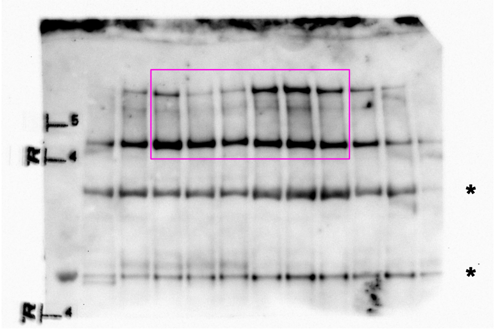

W.B. #9
anti-ApoB 1:1,500
* unspecific bands

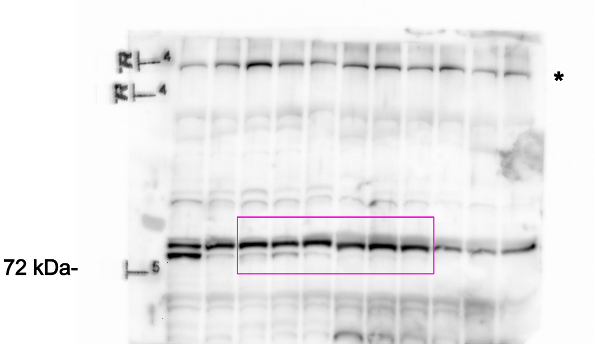

W.B. #10
anti-Calnexin 1:15,000
* unspecific band

Mouse liver heavy membranes (20 µg/lane)

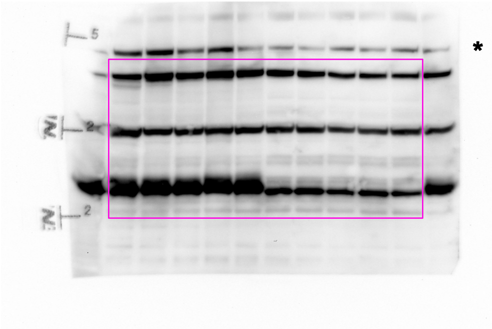

W.B. #11
anti-Calnexin 1:20,000
anti-Calreticulin 1:150,000
anti-ApoE 1:3,000
* unspecific band

Additional file 3

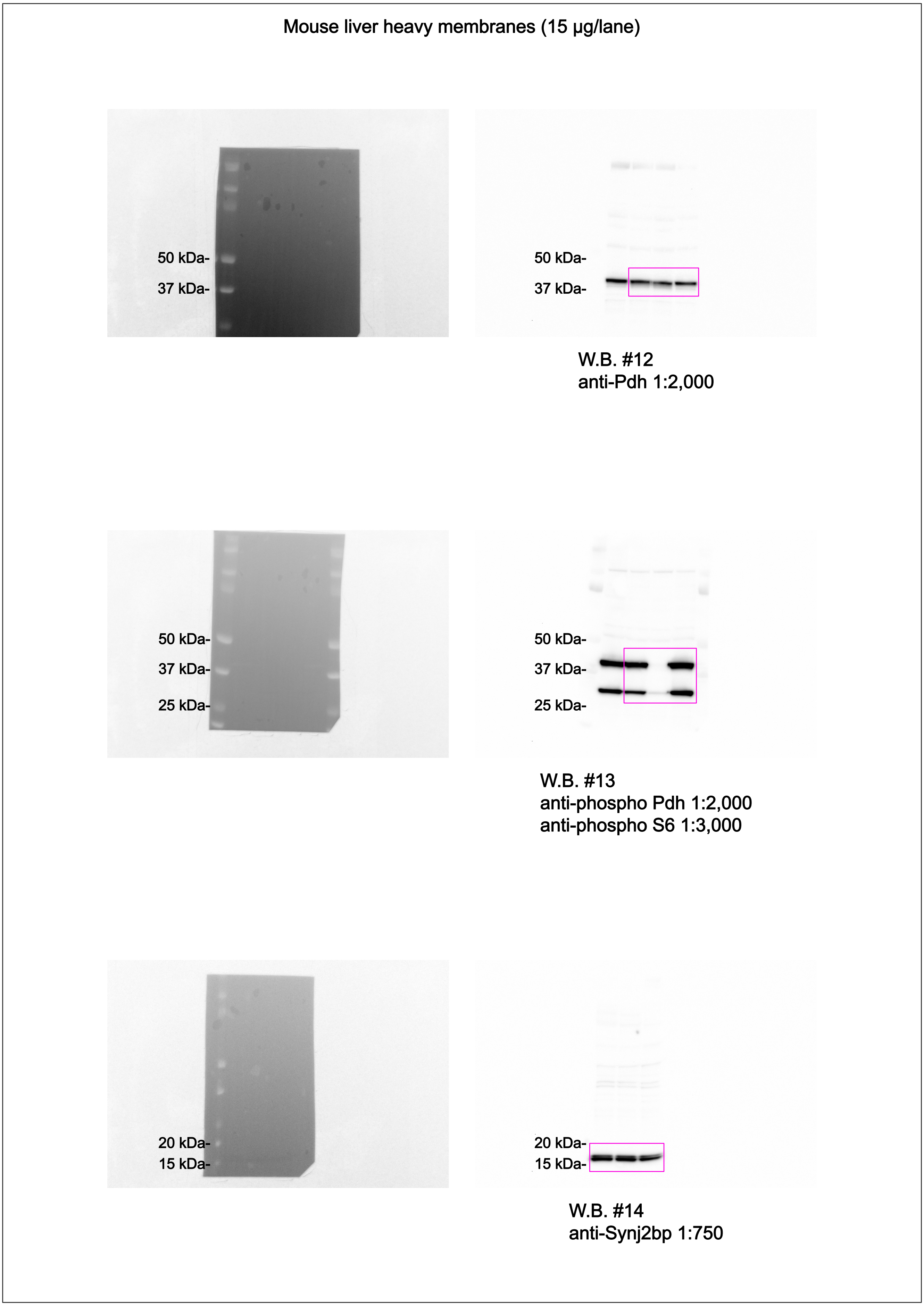

Supplement: Supplementary file 3 — Additional file 3. Original, uncropped immunoblots. [file 13062_2022_344_MOESM3_ESM.pdf]
